# A comprehensive analysis of genes associated with hypoxia and cuproptosis in pulmonary arterial hypertension using machine learning methods and immune infiltration analysis: *AHR* is a key gene in the cuproptosis process

**DOI:** 10.3389/fmed.2024.1435068

**Published:** 2024-09-26

**Authors:** Zuguang Chen, Lingyue Song, Ming Zhong, Lingpin Pang, Jie Sun, Qian Xian, Tao Huang, Fengwei Xie, Junfen Cheng, Kaili Fu, Zhihai Huang, Dingyu Guo, Riken Chen, Xishi Sun, Chunyi Huang

**Affiliations:** ^1^Central People’s Hospital of Zhanjiang, Zhanjiang, Guangdong, China; ^2^Emergency Medicine Center, Affiliated Hospital of Guangdong Medical University, Zhanjiang, Guangdong, China; ^3^Respiratory and Critical Care Medicine, The Second Affiliated Hospital of Guangdong Medical University, Zhanjiang, Guangdong, China

**Keywords:** pulmonary arterial hypertension, bioinformatics analysis, immune infiltration, hub gene, *AHR*, *FAS*, *FGF2*

## Abstract

**Background:**

Pulmonary arterial hypertension (PAH) is a serious condition characterized by elevated pulmonary artery pressure, leading to right heart failure and increased mortality. This study investigates the link between PAH and genes associated with hypoxia and cuproptosis.

**Methods:**

We utilized expression profiles and single-cell RNA-seq data of PAH from the GEO database and genecad. Genes related to cuproptosis and hypoxia were identified. After normalizing the data, differential gene expression was analyzed between PAH and control groups. We performed clustering analyses on cuproptosis-related genes and constructed a weighted gene co-expression network (WGCNA) to identify key genes linked to cuproptosis subtype scores. KEGG, GO, and DO enrichment analyses were conducted for hypoxia-related genes, and a protein–protein interaction (PPI) network was created using STRING. Immune cell composition differences were examined between groups. SingleR and Seurat were used for scRNA-seq data analysis, with PCA and t-SNE for dimensionality reduction. We analyzed hub gene expression across single-cell clusters and built a diagnostic model using LASSO and random forest, optimizing parameters with 10-fold cross-validation. A total of 113 combinations of 12 machine learning algorithms were employed to evaluate model accuracy. GSEA was utilized for pathway enrichment analysis of *AHR* and *FAS*, and a Nomogram was created to assess risk impact. We also analyzed the correlation between key genes and immune cell types using Spearman correlation.

**Results:**

We identified several diagnostic genes for PAH linked to hypoxia and cuproptosis. PPI networks illustrated relationships among these hub genes, with immune infiltration analysis highlighting associations with monocytes, macrophages, and CD8 T cells. The genes *AHR*, *FAS*, and *FGF2* emerged as key markers, forming a robust diagnostic model (NaiveBayes) with an AUC of 0.9.

**Conclusion:**

*AHR*, *FAS*, and *FGF2* were identified as potential biomarkers for PAH, influencing cell proliferation and inflammatory responses, thereby offering new insights for PAH prevention and treatment.

## 1 Introduction

Pulmonary arterial hypertension (PAH) is a rare and serious disease, with an incidence of 15 to 50 cases per million people in the United States and Europe (1). The prevalence is estimated at 4.8 to 8.1 cases per million in children and 5.6 to 25 cases per million in adults (2). The prognosis for PAH is grim, primarily due to progressive elevation of pulmonary artery pressure, culminating in right heart failure and mortality. According to the National Center for Health Statistics, the 1-year survival rate for untreated PAH is 68%, dropping to 48% at 3 years and a mere 34% at 5 years (3). The annual mortality rate of PAH remains approximately 10% even with modern treatment techniques. Clinical manifestations of PAH encompass dyspnea, chest pain, syncope, lower limb edema, and jugular vein distension (4). The diagnosis of PAH poses challenges due to the absence of characteristic clinical manifestations. Diagnosis of PAH involves electrocardiography, echocardiography, and right heart catheterization (RHC), the gold standard diagnostic tool (5). However, RHC may lead to misdiagnosis due to various factors (6), causing diagnostic delays. Delayed diagnosis represents a primary contributor to poor patient prognosis (7). Thus, enhancing diagnostic tools for PAH is imperative. The widespread utilization of gene expression profiling (GEO) data has made bioinformatics analysis a pivotal tool for identifying potential genetic biomarkers in the diagnosis and treatment of PAH.

Hypoxia is a prevalent concomitant symptom of PAH. Dyspnea occurs in approximately 98% of PAH patients, with 60% experiencing it as the initial symptom (4). Hypoxia constitutes a significant risk factor for PAH (8) and serves as a common stimulus for inducing PAH in experimental models (9). A study investigating predictive factors for PAH development in patients with hypersensitivity pneumonitis identified hypoxemia as a predictor (10). Hypoxia is believed to initiate endothelial cell dysfunction in PAH, leading to abnormal proliferation of pulmonary artery vascular endothelial cells, vessel wall thickening, and pressure elevation, thereby fostering PAH development (11, 12).

Copper is an indispensable trace element for human physiology. Imbalances in copper levels have been strongly linked to various diseases, including Menkes disease, Wilson’s disease, neurodegenerative disorders, cancer, and cardiovascular diseases (13). A prospective pilot study conducted at a single center revealed significantly elevated blood copper levels among patients with PAH, suggesting a potential role of elevated copper levels as either a causative factor or a marker for PAH (14). Research indicates that copper plays a significant role in regulating the growth and proliferation of endothelial cells in PAH (15), possibly contributing to the pathogenesis of the condition. Cuproptosis, a novel form of copper-dependent cell death, has emerged as an area of study (13), with research extending to diverse conditions such as hepatocellular carcinoma, diabetes mellitus, glioblastoma, and oral squamous cell carcinoma (16–19). However, there remains a paucity of studies investigating the association between copper and PAH.

Both hypoxia and copper are closely linked to the pathogenesis of PAH, yet the relationship between genes associated with hypoxia and copper-induced cell death and PAH remains inadequately investigated. This study aimed to identify potential diagnostic biomarkers for PAH associated with hypoxia and copper-mediated cell death through bioinformatics analysis.

## 2 Method

### 2.1 Data collection and preprocessing

Transcriptome data for PAH were retrieved from the GEO dataset GSE15197, comprising 13 normal and 26 PAH groups. Additionally, validation was conducted using data from GSE33463, consisting of 41 normal and 72 PAH groups. Dataset GSE113439 was used as validation group 2, including 11 normal groups and 15 PAH groups. Dataset GSE22356 was used as validation group 3, there were 10 normal groups and 18 PAH groups. The gene set related to copper-induced cell death was sourced from the Genecard database and literature, while hypoxia-related genes were obtained from the same database. Furthermore, single-cell 10x data for Single-cell Ribonucleic Acid Sequencing (scRNA-seq) was obtained from the GEO dataset GSE228644. Data preprocessing of the GEO dataset involved normalization using the “normalizeBetweenArrays” function.

### 2.2 Transcriptome analysis

Differential gene expression analysis was performed by comparing the 13 normal and 26 PAH groups in GSE15197, applying criteria of |log Foldchange (FC) | > 1 and a false discovery rate (FDR) < 0.05. Gene set enrichment analysis (GEA) was executed using the GSEA Base R package on genes with a *p*-value >0.05.

### 2.3 Differential gene expression and clustering for copper-induced cell death

Differential analysis was conducted on genes associated with copper-induced cell death, followed by clustering using the “ConsensusClusterPlus” R package to delineate molecular subtypes linked to copper-induced cell death. Additionally, variations in the typed copper-induced cell death genes among subtypes were assessed.

### 2.4 Weighted gene co-expression network analysis of typing results

Weighted gene co-expression network analysis (WGCNA) is a systems biology approach used to delineate patterns of gene associations across different samples. Employing the “WGCNA” R package, we identified genes significantly linked to the cuproptosis subtype score. Initially, we inputted expression profiles from the top 25% of variants in the GSE15197 cohort, excluding samples with cluster heights exceeding 20,000. We established a soft threshold and selected the two modules exhibiting the strongest positive correlations with the cuproptosis subtype score through Pearson correlation coefficient calculation.

### 2.5 Functional enrichment for hypoxia genes

To analyze hypoxia-associated genes, including KEGG pathways, Gene Ontology (GO), and Disease Ontology (DO) enrichment, we utilized the “enrich” function in R.

### 2.6 Protein interaction network analysis and ROC curve plotting

The final hub genes were obtained by intersecting sets of WGCNA genes, PAH differential genes, and hypoxia-associated genes. Subsequently, the protein interaction network of hub genes was scrutinized using the STRING database, with visualization conducted via Cytoscape. Further analysis of the protein network was performed using the MCODE tool. Receiver Operating Characteristic (ROC) analysis of the hub genes was carried out using the “pROC” R package.

### 2.7 Immune landscape analysis and genetic correlation analysis

The CIBERSORT algorithm predicted the composition of infiltrating immune cells in each tumor sample and assessed immune cell differences between normal and disease groups. Subsequently, immune cell profiles associated with hub genes were examined using the infiltration results. Correlations among hub genes were analyzed using the limma package.

### 2.8 Single-cell downscaling and cluster annotation

We analyzed scRNA-seq data using R software packages, including “Seurat” and “SingleR.” To ensure high-quality data, we applied four filters to the raw matrix of each cell: genes expressed in fewer than three cells or more than 10,000 cells, as well as cells with over 20% mitochondrial genes, were excluded. Data normalization was conducted using the “NormalizeData” function in the “Seurat” package, employing the “LogNormalize” method. The normalized data were then processed into Seurat objects, and the top 2000 highly variable genes were identified using the “FindVariableFeatures” function. Principal component analysis (PCA) was performed on the normalized objects using the “RunPCA” function. Subsequently, the dimensionality of the scRNA-seq data was reduced based on the first 2000 genes. The original distributions of the data were visualized using the “RunTSNE” function for t-distribution random neighbor embedding (t-SNE). We utilized the “RunHarmony” function for de-batching and downscaling the data. Significant principal components were identified using JackStraw analysis, and the top 17 components were selected for clustering analysis. Cell clustering was performed using the “FindNeighbors” and “FindClusters” functions in the “Seurat” package. Based on Euclidean distance in PCA, we constructed a k-nearest-neighbor graph using the “FindNeighbors” function and visualized the downscaled resolution using the clustree function. The resolution was set to 1 for combined results. SingleR annotation was integrated with the toppgene database, and cell groups were labeled accordingly.

### 2.9 Analysis of single-cell gene sets

Hub genes underwent differential analysis using the “FindAllMarkers” function. Subsequently, the resulting genes were scored using the “irGSEA.score” function, and the significance of these scores was displayed in each cluster.

### 2.10 Machine learning for constructing diagnostic models

To determine the optimal regularization parameter (*λ*) of the model, we performed 10-fold cross-validation using least absolute shrinkage and selection operator (LASSO) logistic regression. The cv.glmnet function was used to select the value of λ that best explained the data based on the deviance criterion. We plotted the path plots and cross-validation curves of the LASSO regression to visually demonstrate the coefficient shrinkage process and the evaluation of model performance. Finally, we extracted the characteristic genes with nonzero regression coefficients in the LASSO model at the optimal *λ* value, and screened out the significantly associated genes. Similarly, randomForest function was used to construct a random forest model, and the model parameter was set to construct 500 decision trees (ntree = 500). We generated a plot of the error rate of the random forest model (forest.pdf) to evaluate the stability and accuracy of the model. Subsequently, we re-constructed the random forest model by cross-validating the number of trees that selected the least error to improve model performance. The MeanDecreaseGini (importance score) of genes was extracted by the importance function, and genes with a score greater than 2 were screened as significantly contributing to classification. Ultimately, genes that overlap in the two machine-learning algorithms are considered diagnostic biomarkers.

We combined 12 machine learning algorithms to generate 113 algorithm combinations to further screen for consistent regulatory genes (CRGs) with good accuracy and stability. Ensemble algorithms include random forest (RF), least absolute shrinkage and selection operator (Lasso), Ridge, elastic network (Enet), Stepglm, support vector machine (SVM), glmBoost, linear discriminant analysis (LDA), gradient boosting machine (GBM), extreme gradient boosting machine (XGBoost), and Bayesian method. GSE15197 and GSE33463 were merged into the training group, and GSE113439 and GSE22356 were used as the test group. The fitted diagnostic model was constructed based on the 10-fold cross-validation of the train dataset. For each model, the area under receiver operating characteristic curve (AUC) value was determined in all validation datasets, and the model with the highest mean AUC value was considered to be the optimal. ROC curves of the three data sets were constructed for each biomarker.

### 2.11 Characterization-based analysis of GSEA enrichment of genes

Pathways of *AHR* enrichment in high and low expression groups were analyzed via Gene Set Enrichment Analysis (GSEA) to elucidate potential mechanisms. The reference gene set comprised c2.cp.kegg.Hs.symbols.gmt and c5.go.Hs.symbols.gmt, with a screening condition of *p*-value <0.05.

### 2.12 Nomogram construction and immunoanalysis of genes

To further evaluate the validity of the model, a Nomogram was constructed to show the influence of each variable on disease risk. To verify the stability and prediction accuracy of the model, calibration curves were generated using Bootstrapping (1,000 replicates). Finally, decision curve analysis (DCA) was used to evaluate the clinical applicability of the model under different risk thresholds. All statistical analyses were performed in the R language environment using the rms and rmda packages. We then further analyzed the infiltration of key genes from the proportion of immune cells analyzed by CIBERSORT. Spearman correlation analysis was used to assess the correlation between different immune cell types and *AHR* and *FAS* expression (20–22).

### 2.13 Time series analysis of Mfuzz and functional enrichment

Time-series analysis of all sample genes by comparing *AHR* expression was performed using the MfuzzR package, and they were divided into 50 categories. The most valuable category was obtained by calculating the correlation between each cluster and *AHR*, and then functional and pathway clustering analysis was performed on these categories. Finally, using the http://bar.utoronto.ca/efp_human/cgi-bin/efpWeb.cgi online analysis of *AHR* and location of the *FAS*.

## 3 Results

### 3.1 Differential analysis of transcriptome data and GSEA enrichment analysis

**Figure 1:**
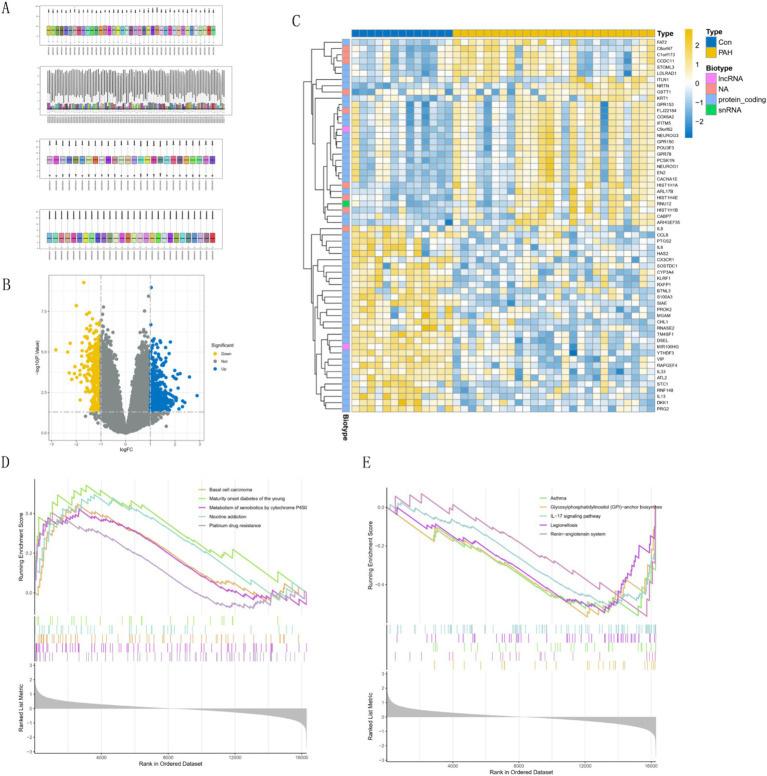
Comprehensive gene expression analysis. **(A)** Sample normalization process. **(B)** Volcano plots of differentially expressed genes (DEGs). Yellow is up-regulated in the Depression Group, and blue is up-regulated in the control group. **(C)** Heatmap of top 30 genes. **(D,E)** Gene set enrichment analysis (GSEA) of differential genes.

Figure 1A shows the normalization of the four PAH datasets. We identified 225 up-regulated genes and 366 down-regulated genes through differential analysis of corrected GSE15197 data between pulmonary arterial hypertension (PAH) and normal lung tissues (Figure 1B). Additionally, we examined the expression of the top 30 genes with the most significant differences between PAH and normal controls (Figure 1C). Analysis of the GSEA results revealed that the five most significantly enriched pathways for downregulated genes encompassed Basal cell carcinoma, Maturity onset diabetes of the young, Metabolism of xenobiotics by cytochrome P450, Nicotine addiction, and Platinum drug resistance (Figure 1D). Conversely, the five most significantly up-regulated genes were enriched in pathways including Glycosylphosphatidylinositol (GPI)-anchor biosynthesis, Asthma, IL-17 signaling pathway, Legionellosis, and Renin-angiotensin system (Figure 1E).

### 3.2 Identification and typing of cuproptosis-related genes and WGCNA analysis

**Figure 2:**
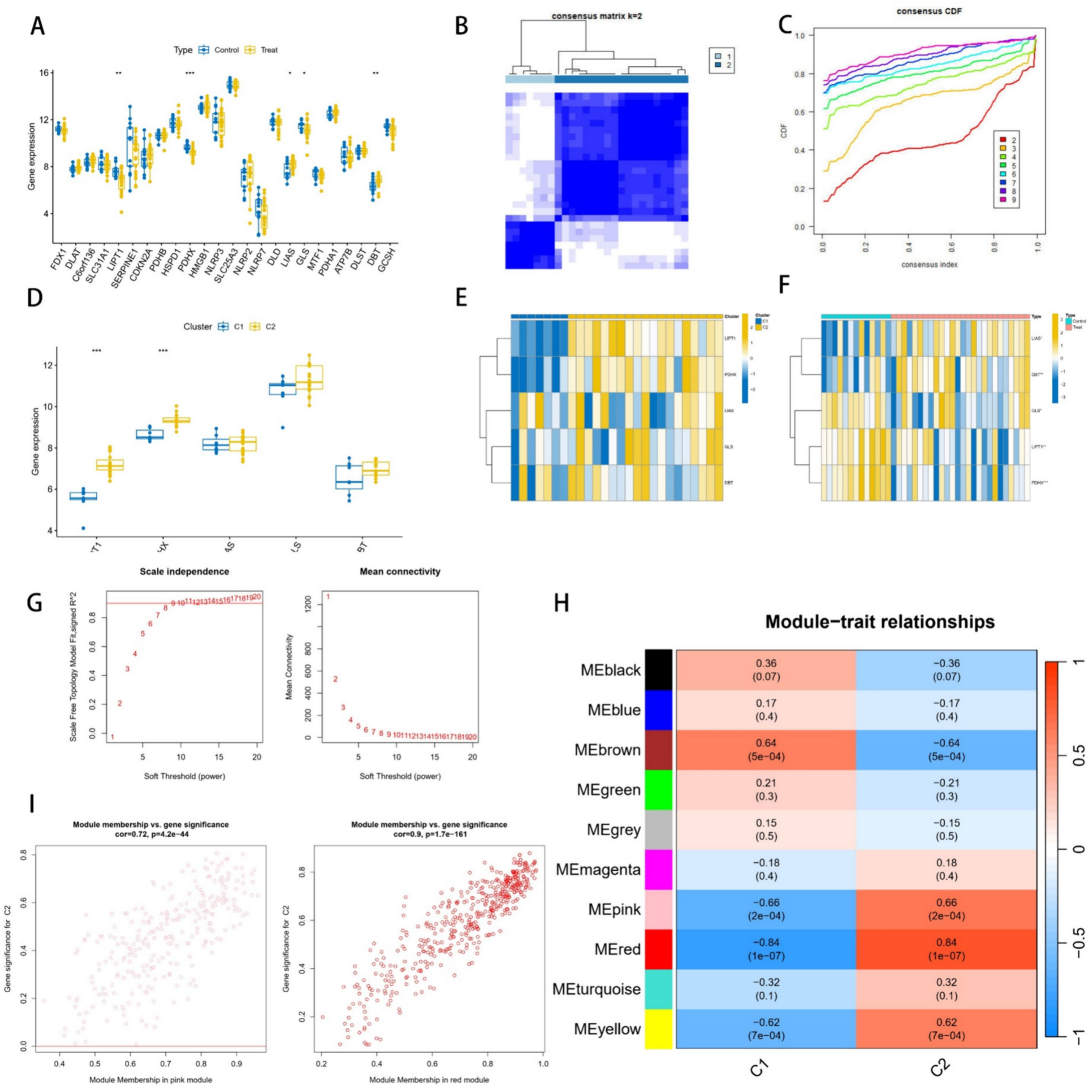
Analysis of cuproptosis genes in pulmonary arterial hypertension (PAH). **(A)** Expression of cuproptosis genes in PAH and normal groups. **(B)** Consistent clustering matrix at k = 2. **(C)** Consensus clustering cumulative distribution function **(C,D,F)** for k = 2 to k = 9. **(D)** Expression of cuproptosis genes in two subtypes. **(E,F)** Heatmap of cuproptosis signature genes. **(G)** Network analysis. Left: Scale-Free Fit Index versus Soft Threshold Power. Right: Mean Connectivity as a Function of Soft Threshold Power. **(H)** Pearson correlation analysis of merged modules in two subtypes. Each row in the heatmap corresponds to a module eigengene (ME), and each column corresponds to a clinical feature. **(I)** Correlation analysis of feature genes in pink and red modules.

Based on the literature and Genecard database, we obtained 25 cuproptosis-related genes, of which five genes—*LIPT1*, *PDHX*, *LIAS*, *GLS*, and *DBT*—showed differential expression in the transcriptome (Figure 2A). Unsupervised cluster typing of GSE15197 using these five differential cuproptosis-related genes segregated them into two subgroups (Figures 2B,C), with Subtype II displaying stronger association with cuproptosis, as evidenced by gene variance analysis (Figure 2D). Figures 2E,F depict the differential expression of the five genes across various clusters and groups. WGCNA was employed to further investigate genomes associated with these subtypes. When the soft threshold value was set to 8, the data exhibited consistency with the power law distribution, and the average concatenation appeared stable (Figure 2G), rendering it suitable for subsequent analysis. Ultimately, we identified 10 modules (Figure 2H), with comparative analysis revealing that the MEred (cor = 0.9, *p* = 1.7e-161) and MEpink (cor = 0.72, *p* = 4.2e-44) modules were most closely correlated with subtype II (Figure 2I), encompassing 712 genes.

### 3.3 Functional analysis of hypoxia target genes and PPI construction of disease genes

**Figure 3:**
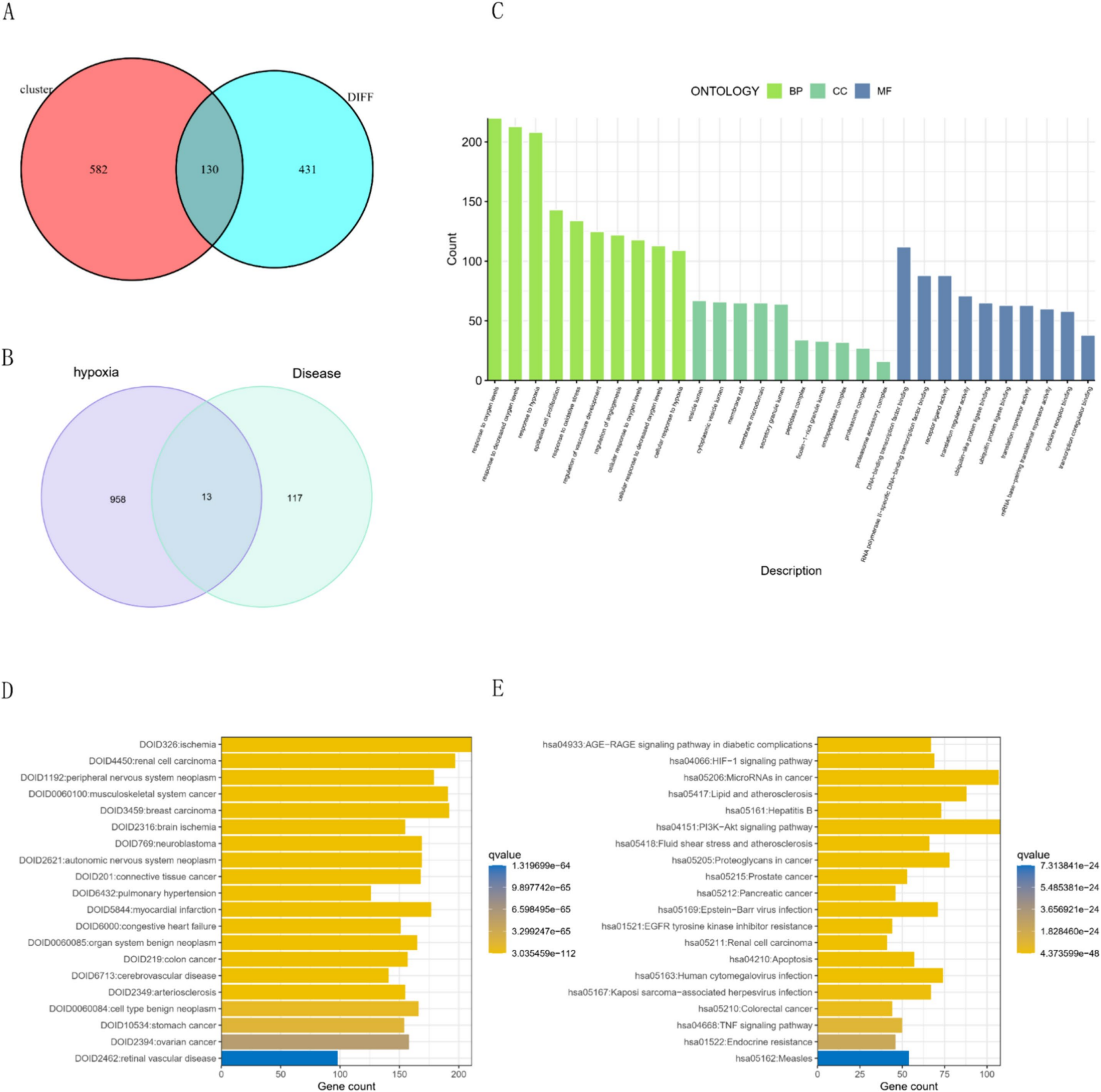
Gene intersections and enrichment analyses in hypoxia-related conditions. **(A)** Intersection of modular and differential genes. **(B)** Intersection of disease genes and hypoxia targets. **(C)** GO enrichment analysis for hypoxic targets. Presentation of the top five results for BP, CC, and MF. **(D)** Disease ontology (DO) enrichment results. The top 20 enriched disease terms were displayed. **(E)** KEGG pathway enrichment results: Top 20 enriched pathways were displayed.

The intersection of key genes from two modules and differential genes from the transcriptome analysis resulted in 130 disease genes (Figure 3A). We retrieved 972 genes from Genecard with a correlation score > 0.2, and the overlap between the hypoxia gene set and disease genes identified 13 hub genes (Figure 3B). Functional enrichment analysis of hypoxia-related genes revealed Gene Ontology (GO) results indicative of responses to oxygen level changes, hypoxia, and cellular components such as vesicle lumen and membrane rafts (Figure 3C). Disease Ontology (DO) enrichment highlighted significant associations with PAH, ischemia, renal cell carcinoma, and peripheral nervous system tumors (Figure 3D). Additionally, Kyoto Encyclopedia of Genes and Genomes (KEGG) analysis indicated significant enrichment in pathways including the HIF-1 signaling pathway, microRNA in cancer, and AGE-RAGE signaling pathway in diabetic complications (Figure 3E).

**Figure 4:**
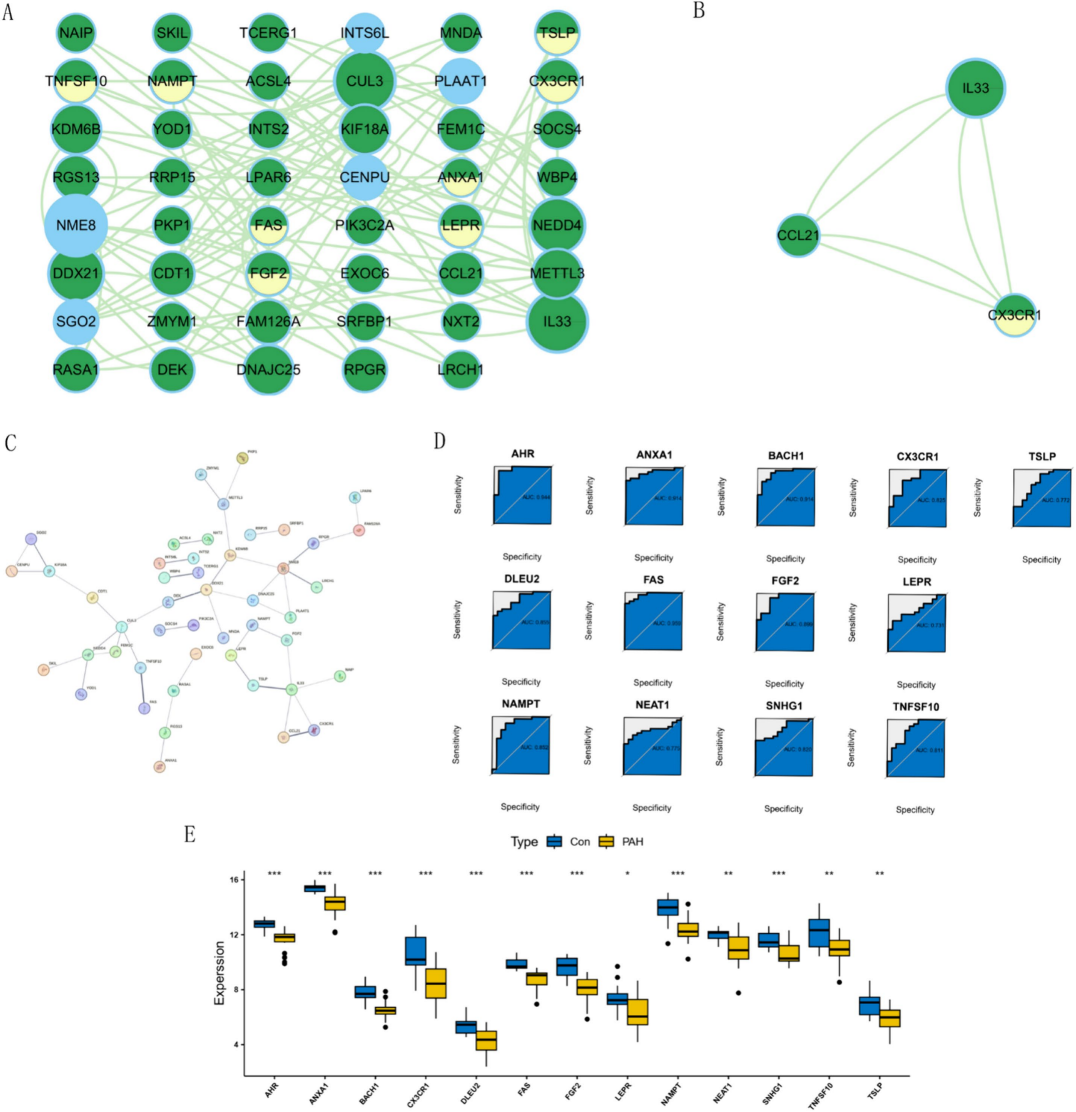
Network analysis and gene expression profiling in PAH. **(A)** Visualization of disease targets in cytoscape. **(B)** Result of the MCODE algorithm. **(C)** Original Protein–Protein Interaction (PPI) Network. **(D)** ROC curves for each hub gene. **(E)** Expression of hub Genes in PAH and normal groups.

We employed the STRING database to construct a protein–protein interaction (PPI) network, where green nodes represented disease genes, blue nodes represented genes from STRING, and yellow nodes represented hub genes. This network analysis revealed direct and indirect regulatory targets of the hub genes (Figure 4A). Utilizing the MCODE algorithm, we identified the *CCL21-IL33-CX3CR1* cluster, with *CX3CR1* identified as the hub gene, suggesting potential indirect regulation of *CCL21* and *IL33* in PAH development (Figure 4B). Additionally, raw PPI network images were generated (Figure 4C). ROC curve analysis demonstrated that all hub genes had an AUC above 0.7, with *FAS* exhibiting the highest AUC value of 0.959 (Figure 4D). Differential expression analysis indicated that all hub genes were highly expressed in the normal group, suggesting their role as protective genes (Figure 4E).

### 3.4 Immune infiltration and immune differential analysis

**Figure 5:**
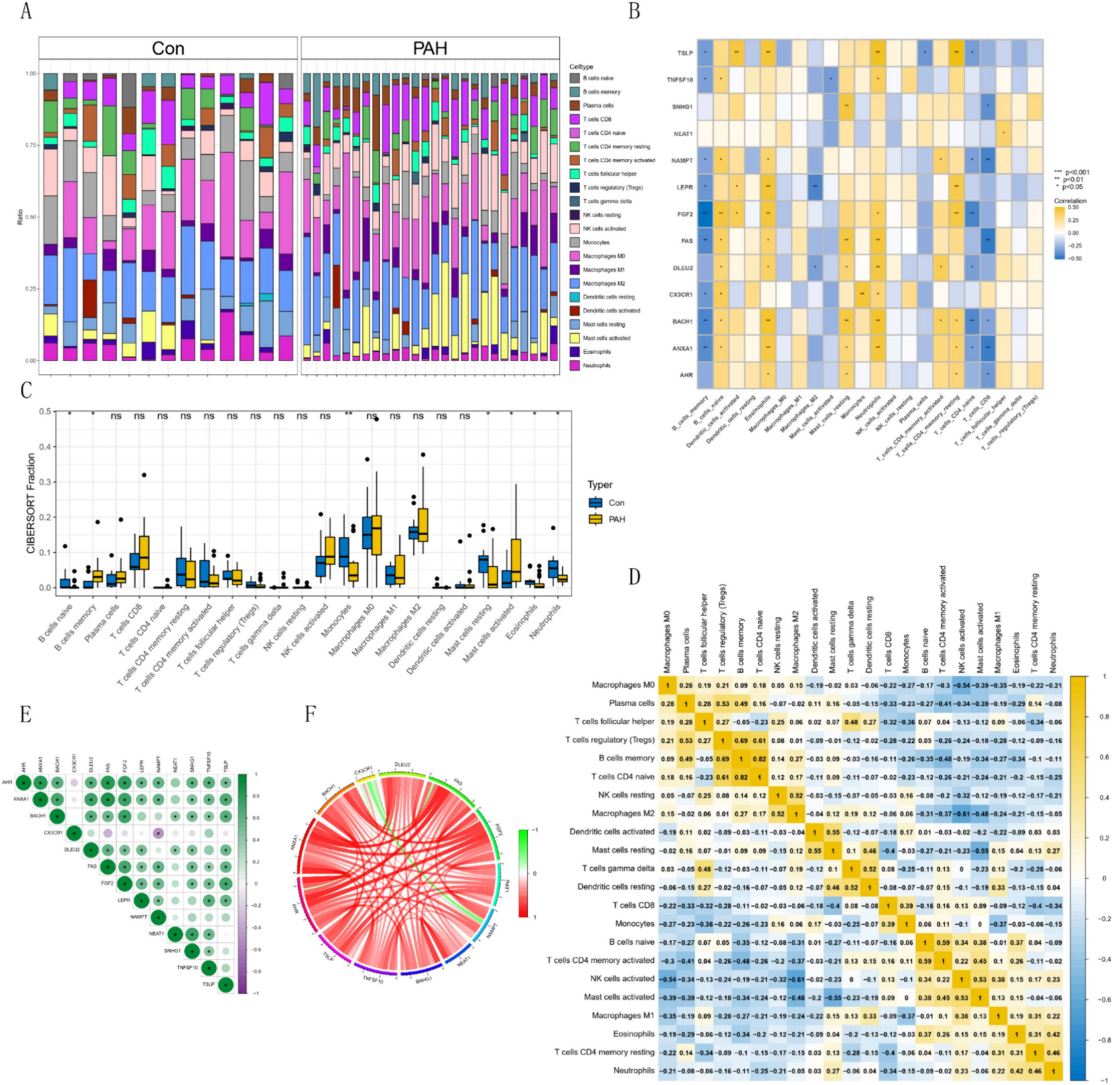
Immune cell distribution and correlations in GSE15197 dataset. **(A)** Relative proportion of immune cell infiltrates. **(B)** Correlation analysis of hub genes with immune cells. **(C)** Comparison of 22 immune cell types in PAH and normal groups. Blue indicates normal and yellow indicates PAH groups. **(D)** Correlations among 22 different immune cell populations: Yellow and blue indicate positive and negative correlations, respectively, and white indicates no correlation between the indicated immune cell populations. **(E,F)** Correlation analysis of hub genes.

Using the CIBERSORT algorithm, we assessed immune infiltration in GSE15197 and visualized the immune cell proportions for each sample (Figure 5A). Based on these results, the hub gene was significantly under-expressed in memory B cells and over-expressed in naive B cells, with significant differences also observed in memory resting CD4+ T cells, naive CD4+ T cells, and CD8+ T cells (Figure 5B). Differential analysis of immune cells between normal and tumor groups revealed superior immune cell infiltration in the normal group, particularly evident in monocytes, naive B cells, and neutrophils, while activated mast cells and memory B cells were significantly infiltrated in tumors (Figure 5C). Furthermore, correlation analysis among various immune cells revealed distinct expression patterns (Figure 5D). Analyzing hub gene correlations, *CX3CR1* and *NAMPT* exhibited significant negative correlations, while other genes showed significant positive correlations. *AHR* displayed positive correlations with *ANXA1*, *BACH*, *DLEU2*, *FAS*, *FGF2*, *LEPR*, *NAMPT*, *SNHG1*, *TNFSF10*, and *TSLP* (Figures 5E,F).

### 3.5 Single-cell outcome analysis

**Figure 6:**
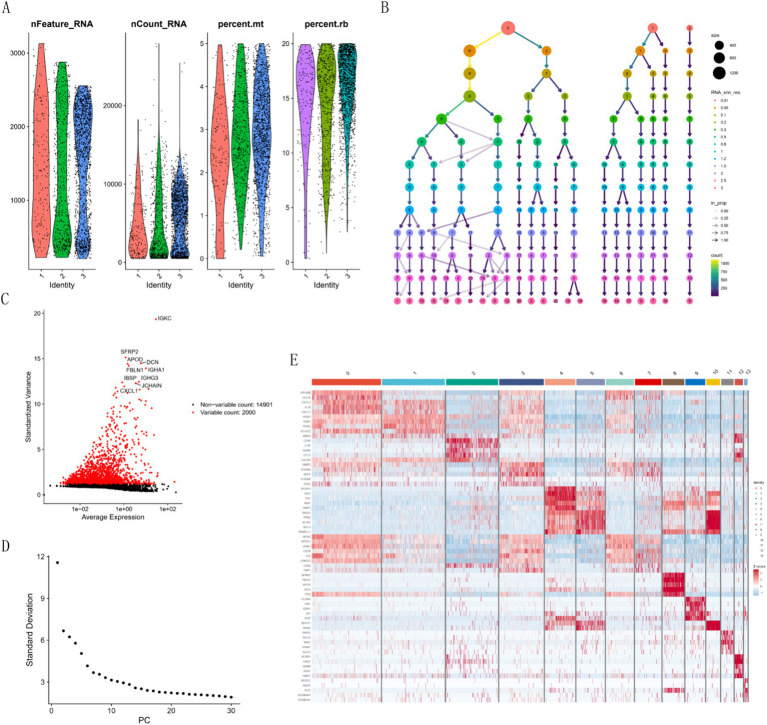
Cell quality control and clustering analysis. **(A)** Histogram of cell quality control results. **(B)** Cluster tree across different resolutions. **(C)** Characterization of top 2,000 significant genes. **(D)** Elbow plot for quantification inflection. **(E)** Differential heatmap of each cell cluster.

**Figure 7:**
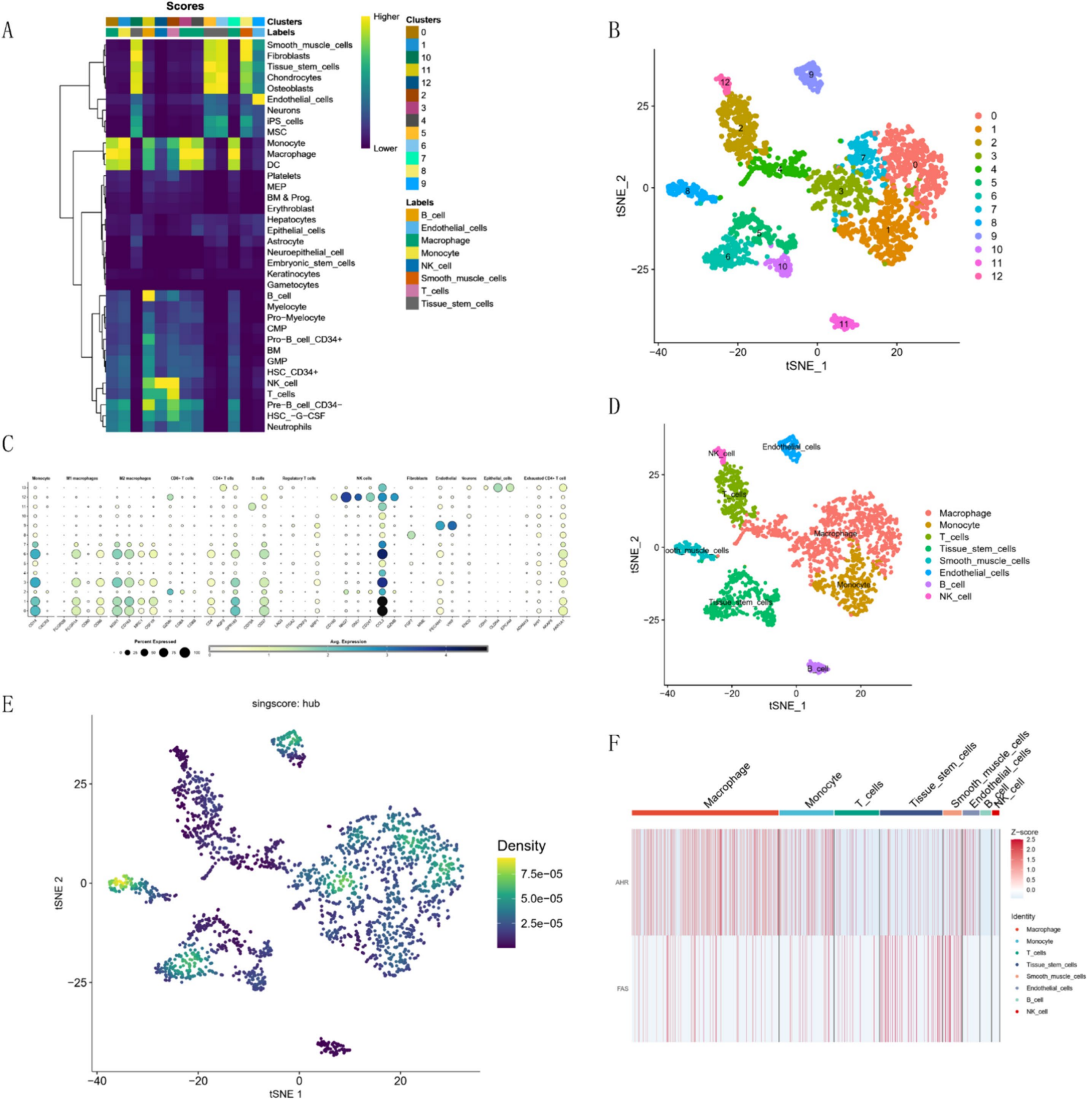
Cell clustering and marker expression analysis. **(A)** Singler’s annotation results. **(B)** t-SNE plot of 13 cell clusters. **(C)** Expression of characteristic marker targets in various cell populations. **(D)** t-SNE plot of cell annotation results. **(E)** AUC-Cell score result graph. **(F)** Z-Score expression of hub genes in each cluster.

We processed the scRNA-seq data from GSE228644, ensuring sample quality by removing specific cells and controlling the mitochondrial and erythroid gene ratio (Figure 6A). Figure 6B is a single-cell cluster tree. Subsequently, we identified 2000 genes exhibiting high variability and highlighted the top 10 most important genes (Figure 6C). Using PCA, we selected 17 principal components (Figure 6D), with all highly variable genes marked in red. We employed the top 1,500 variable genes for principal component analysis to facilitate dimensionality reduction. Based on the findings presented in Figure 6B, a resolution of 1 was selected, and the differential gene expression results within each cluster were examined (Figure 6E) We employed singleR for preliminary annotation of the cell population (Figure 7A). Cell cluster results identified a total of 13 cell clusters (Figure 7B). Figure 7C we observed the enrichment of some common cell characteristic genes in each cell cluster. Cell identity was annotated by combining singleR results with cell characterization genes (Figure 7D), broadly categorized into nine clusters: Macrophage, Monocyte, T cells, Tissue stem cells, Smooth muscle cells, Endothelial cells, B cell, NK cell, and Epithelial cells. Using the results of z-score, it was found that there was significant enrichment on Macrophage, Monocyte, Smooth muscle cells (Figure 7E). The characteristic genes of each cell cluster were obtained by differential analysis (*p* < 0.05). Among them, the heat map of the distribution of key genes *AHR* and *FAS* is shown in Figure 7F.

### 3.6 Identification and validation of diagnostic biomarkers

**Figure 8:**
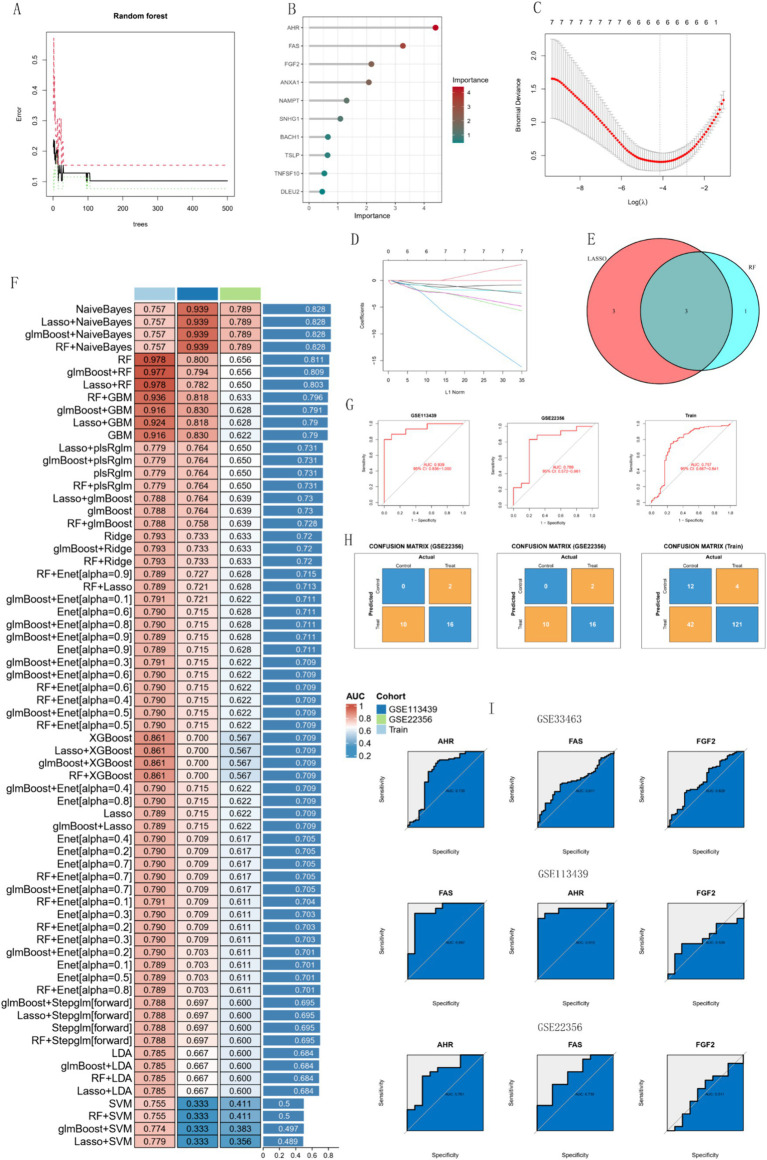
Analysis of biomarkers using machine learning algorithms. **(A,B)** RF algorithm. **(C,D)** Lasso regression analysis. **(E)** Venn diagram showing reliable biomarkers between LASSO and RF. **(F)** Heatmap of models constructed by various machine learning algorithms. **(G)** ROC curves of the models in each dataset. **(H)** Confusion matrices for the models across each dataset. **(I)** ROC curves of the three models’ genes in different test datasets.

Four diagnostic genes were identified using RF as potential diagnostic markers (Figures 8A,B). Using the LASSO regression algorithm, six genes from the selected modules were identified as potential diagnostic biomarkers (Figures 8C,D). Superposition of the results of the two sets of machine-learning algorithms yielded three genes (Figure 8E), including *AHR*, *FAS*, and *FGF2*. *AHR* and *FAS* are the most effective diagnostic markers. On the basis of retaining three genes, the combination of multiple machine learning algorithms obtained 67 models, among which NaiveBayes had the best comprehensive performance in the training group and the two test groups (Figure 8F), with AUC values of 0.939 in GSE113439 and 0.789 in GSE22356, respectively. 0.757 in the train group (Figure 8G). Figure 8H shows the confusion matrix for the three data sets. The AUC values of single genes are shown as shown in Figure 8I, which shows that *AHR* and *FAS* perform best in the three datasets. The expression of the three genes in other datasets is shown in Figures 1–3. In summary, we defined them as hub important genes.

### 3.7 Nomogram construction and immune cell analysis of key genes

**Figure 9:**
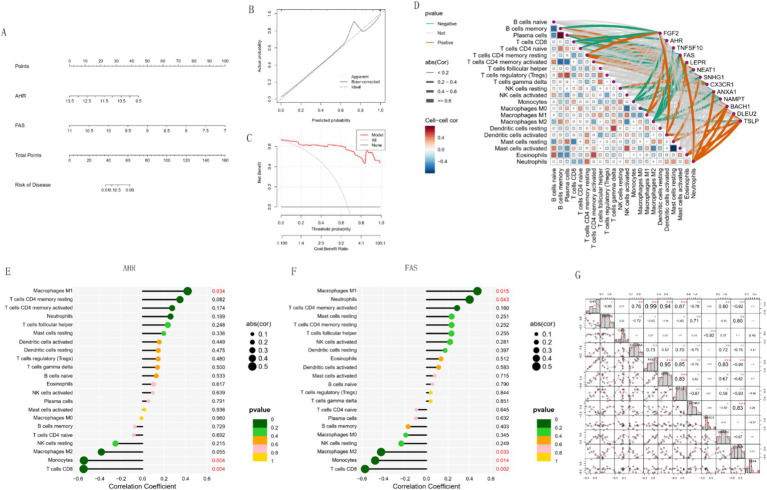
Risk prediction and regulatory relationships analysis. **(A)** Nomogram constructed for risk prediction. **(B,C)** Calibration curve and DCA curve of the nomogram. **(D)** Heatmap showing the regulatory relationships between immune cells and hub genes. **(E,F)** Bar charts illustrating the regulatory relationships between *AHR*, *FAS*, and immune cells. **(G)** Heat map of correlation between key genes and other PAH diagnostic genes.

In this study, we developed a predictive model based on *AHR* and *FAS* expression levels and used a nomogram to estimate disease risk (Figure 9A). The nomogram showed that *AHR* and *FAS* levels were associated with disease risk. The calibration curve of the model (Figure 9B) showed good agreement between the predicted probabilities and the actual results, indicating the high reliability of the model. Decision curve analysis (Figure 9C) further validated the clinical usefulness of the model, providing a net benefit across a range of threshold probabilities.

In addition, correlation network analysis (Figure 9D) revealed complex relationships between immune cell types and gene expression levels. *AHR* and *FAS* were significantly associated with a variety of immune cells, suggesting their potential role in regulating immune responses. Detailed correlation analysis showed that *AHR* was significantly positively correlated with Macrophages M1 (*p* = 0.034) and negatively correlated with Monocytes (*p* = 0.005) and CD8 T cells (*p* = 0.004) (Figure 9E). *FAS* was positively correlated with Macrophages M1 (*p* = 0.015) and Neutrophils (*p* = 0.043). However, there was a significant negative correlation with Macrophages M2 (*p* = 0.033), Monocytes (*p* = 0.014) and T cells CD8 (*p* = 0.002) (Figure 9F). In the correlation matrix heat map (Figure 9G), *AHR*, *FGF2* and *FAS* showed varying degrees of correlation in their relationships with other genes, and *FGF2* was particularly significant in positive correlation, indicating that it may play an important role in the diagnosis of PAH. These results highlight the possible impact of *AHR* and *FAS* in immune cell dynamics and immune-related diseases.

### 3.8 GSEA analysis of the biomarker *AHR*

**Figure 10:**
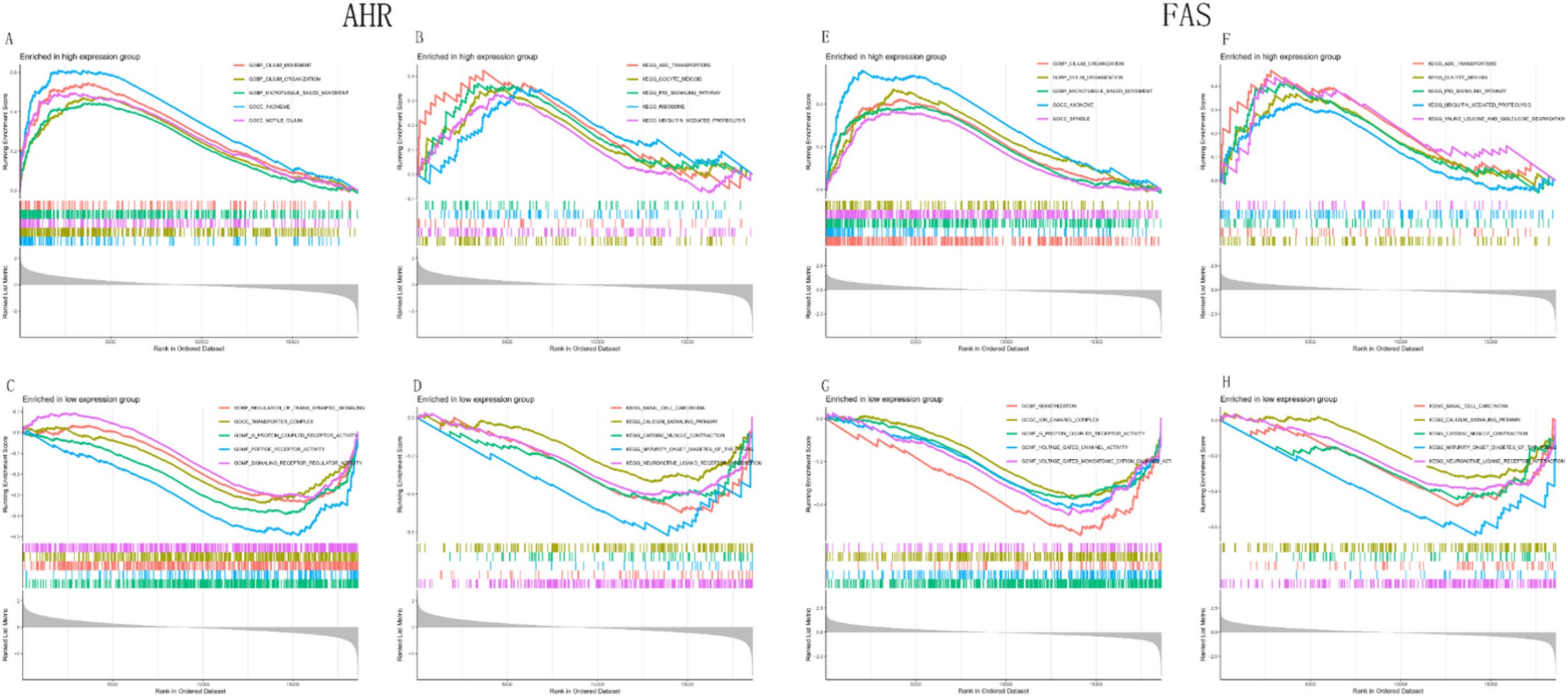
GSEA analysis of functional and pathway enrichment based on gene expression levels. **(A,B)** GSEA analysis of functions and pathways based on high expression of *AHR*. **(C,D)** GSEA analysis of functions and pathways based on low expression of *AHR*. **(E,F)** GSEA analysis of functions and pathways based on high expression of *FAS*. **(G,H)** GSEA analysis of functions and pathways based on low expression of *FAS*.

GSEA results showed that the *AHR* high expression group was enriched in CILIUM_MOVEMENT, CILIUM_ORGANIZATION, MICROTUBULE_BASED_MOVEMENT, etc. (Figure 10A). The pathways in the *AHR* high expression group were enriched in ABC_TRANSPORTERS, OOCYTE_MEIOSIS, P53_SIGNALING_PATHWAY, and others (Figure 10B). The functions in the low expression group were enriched in REGULATION_OF_TRANS_SYNAPTIC_SIGNALING, TRANSPORTER_COMPLEX, G_PROTEIN_COUPLED_RECEPTOR_ACTIVITY, etc. (Figure 10C). Pathways in the *AHR* low expression group were enriched in BASAL_CELL_CARCINOMA, BASAL_CELL_CARCINOMA, CARDIAC_MUSCLE_CONTRACTION, and others (Figure 10D). Similarly, functions in the *FAS* high expression group were enriched in CILIUM_ORGANIZATION and GOLGI_ORGANIZATION, MICROTUBULE_BASED_MOVEMENT (Figure 10E); The pathways in the *FAS* high expression group were enriched in ABC_TRANSPORTERS, OOCYTE_MEIOSIS, P53_SIGNALING_PATHWAY, and others (Figure 10F); In the *FAS* low expression group, functions were enriched in KERATINIZATION, ION_CHANNEL_COMPLEX, G_PROTEIN_COUPLED_RECEPTOR_ACTIVITY, etc. (Figure 10G). In the *FAS* low expression group, the pathways were enriched in BASAL_CELL_CARCINOMA and CALCIUM_SIGNALING_PATHWAY, CARDIAC_MUSCLE_CONTRACTION (Figure 10H).

### 3.9 Mfuzz expression pattern clustering, functional analysis of key modules, and EFP analysis of *AHR*

**Figure 11:**
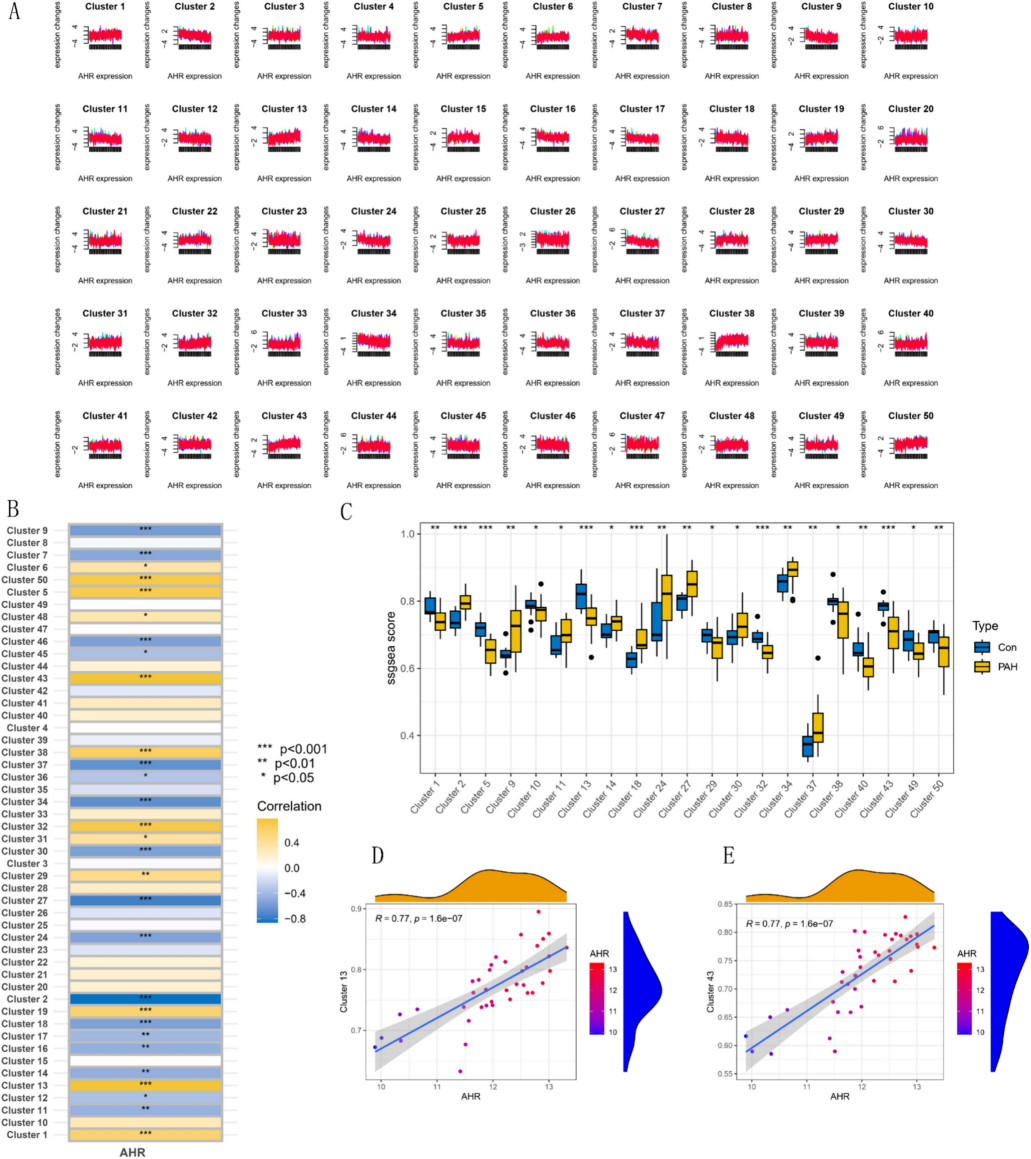
Expression pattern clustering and correlation analyses involving *AHR*. **(A)** Mfuzz expression pattern clustering results. **(B)** Correlation between clustering module and *AHR*. **(C)** SSGSEA clustering module scores and expression characteristics between the PAH group and the normal group. **(D,E)** Correlation analysis of modules 13 and 43 with *AHR*.

**Figure 12:**
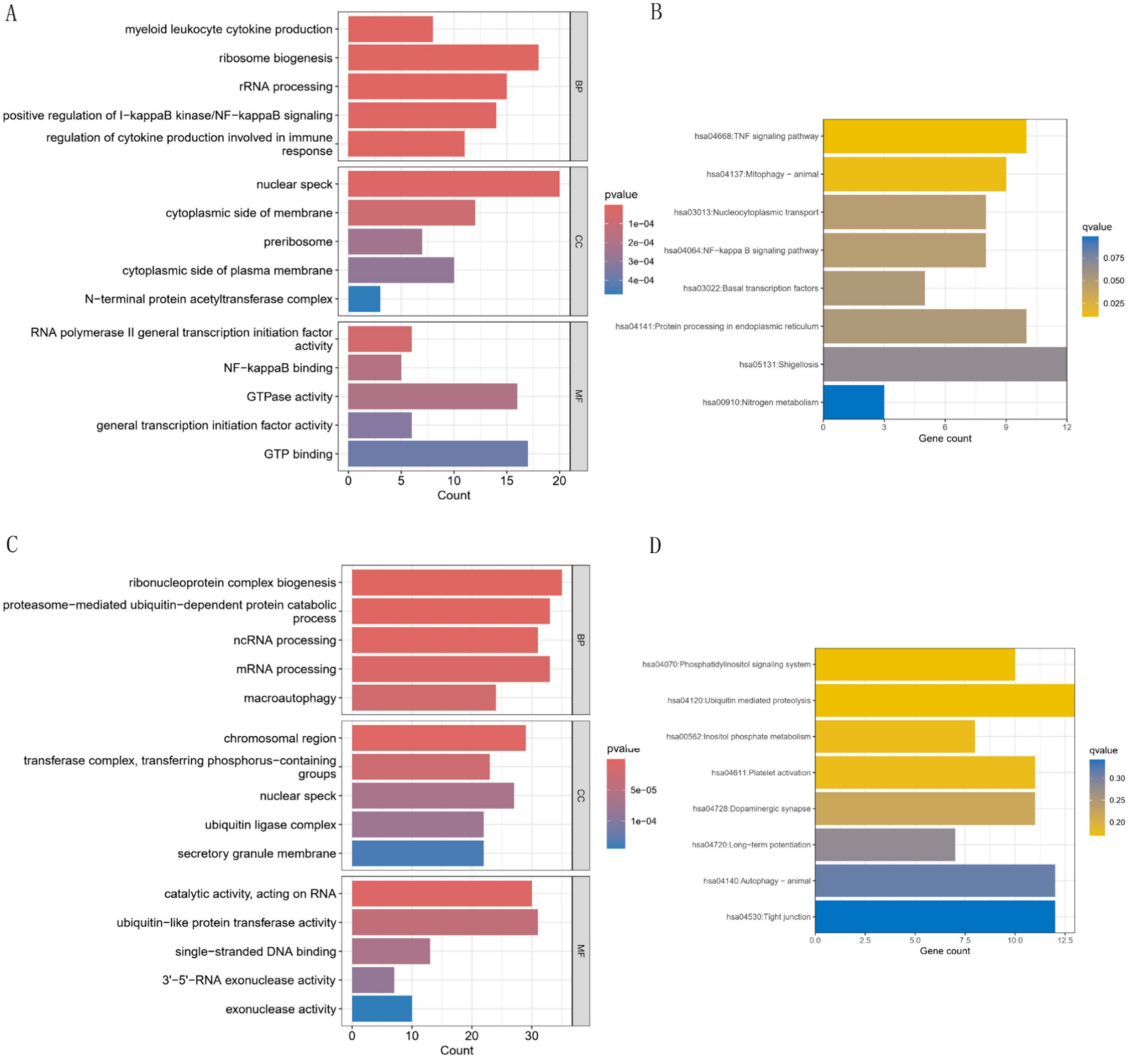
GO and KEGG analyses of characteristic genes in modules 13 and 43. **(A,B)** GO and KEGG analysis for module 13. GO analysis shows the first five results of BP, CC and MF, and KEGG analysis shows the first eight results. **(C,D)** GO and KEGG analysis for module 43. GO analysis shows the first five results of BP, CC and MF, and KEGG analysis shows the first eight results.

**Figure 13:**
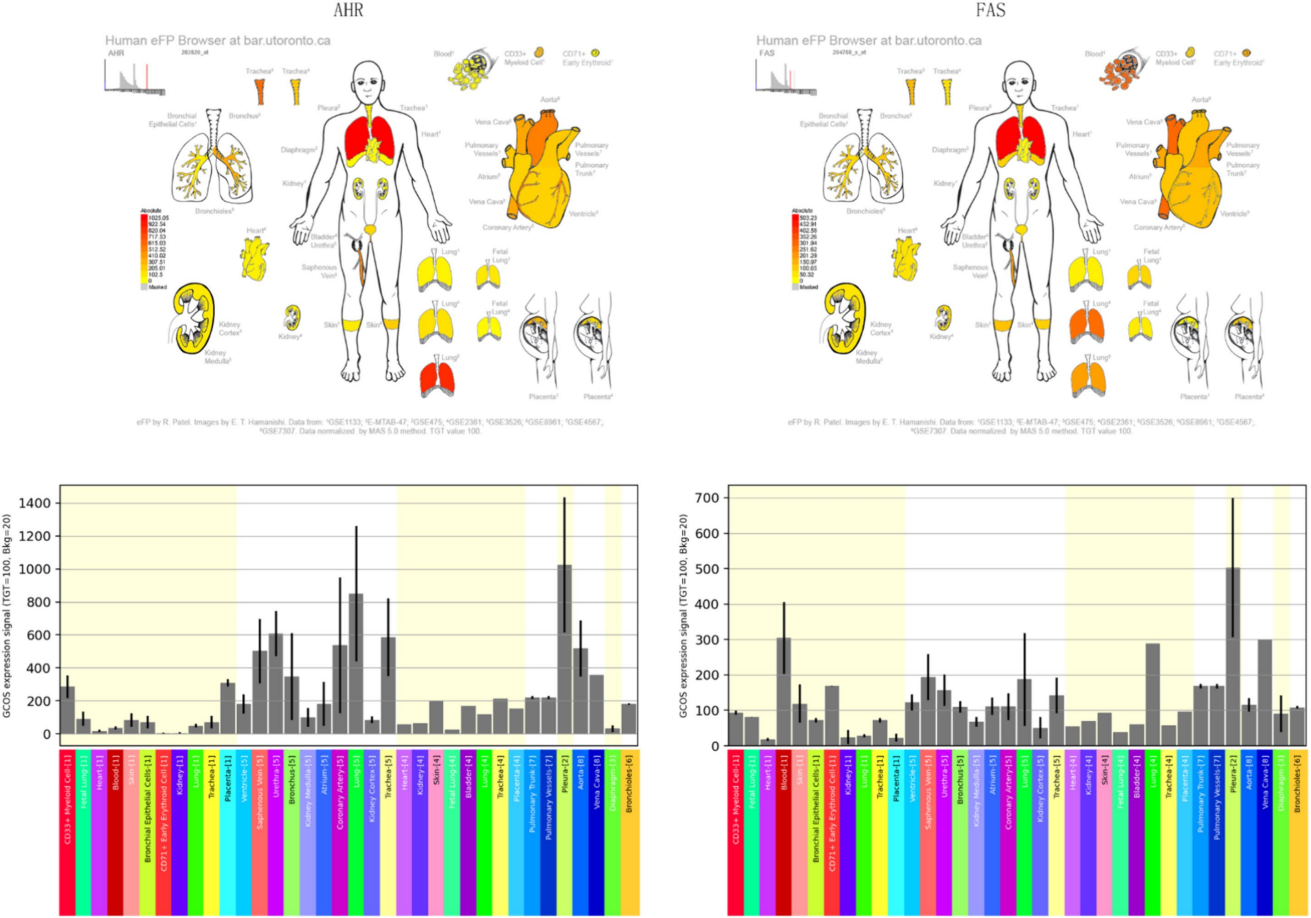
Expression profiles of *AHR* and *FAS* in human tissues obtained using the EFP Tool. Bar charts displaying the expression levels of *AHR* and *FAS* across various human tissues and organs.

Mfuzz analysis yielded 50 clustering results (Figure 11A). Key modules with significant differences between PAH and normal groups, as well as their correlation with *AHR*, were determined based on ssGSEA scoring. Modules 13 and 43 emerged as key modules, positively correlated with *AHR* expression levels (Figures 11B–E). GO enrichment analysis of module 13 genes revealed associations with myeloid leukocytes, cytokine production, and RNA polymerase II general transcription initiation factor activity (Figure 12A), while KEGG enrichment identified relevance to the tumor necrosis factor (TNF) signaling pathway and nucleoplasmic translocation (Figure 12B). Module 43 genes were enriched in functions like ribonucleoprotein complex biogenesis and catalytic activity on RNA, with KEGG enrichment indicating involvement in the phosphatidylinositol signaling system and ubiquitin-mediated proteolysis (Figures 12C,D). Expression Atlas (EFP) analysis demonstrated high *AHR* expression in lung tissues, particularly in the pleura outside lung tissue (Figure 13). EFP results showed that *AHR* was highly expressed in lung tissue, and the bar chart showed that *AHR* was highly expressed in a variety of tissues (organs), with the most significant expression in pleura except lung tissue. Similarly, *FAS* was highly expressed in lung tissue.

## 4 Discussion

PAH presents a severe, life-threatening condition marked by a gradual elevation in pulmonary artery pressure, culminating in right heart failure and mortality. Hypoxia stands out as a significant risk factor for this condition, while copper-mediated cell death represents a novel mode of cell demise, both intricately linked to PAH development. Research has demonstrated the pivotal role of endothelial cell dysfunction in this process, with hypoxia and copper influencing PAH development by regulating endothelial cell proliferation. Therefore, identifying gene markers associated with hypoxia and copper-mediated cell death is imperative for PAH diagnosis. In this study, we explored the relationship between hypoxia- and copper-related genes and PAH, alongside the associated immune infiltration characteristics, through bioinformatics analysis.

Initially, we conducted a differential analysis comparing PAH with normal lung tissues, revealing 225 up-regulated genes and 366 down-regulated genes. Gene Set Enrichment Analysis (GSEA) highlighted the top five enriched pathways for up-regulated genes, including Glycosylphosphatidylinositol (GPI)-anchor biosynthesis, Asthma, IL-17 signaling pathway, Legionellosis, and Renin-angiotensin system. Conversely, the most enriched pathways for down-regulated genes encompassed Basal cell carcinoma, Maturity onset diabetes of the young, Metabolism of xenobiotics by cytochrome P450, Nicotine addiction, and Platinum drug resistance. Notably, the up-regulated pathways are closely linked to inflammation, vasoconstriction, and cell proliferation. The pathogenesis of PAH involves various physiological processes, such as endothelial dysfunction, smooth muscle migration and proliferation, endothelial-mesenchymal transition, inflammation, hypoxia, DNA damage, and oxidative stress (20). Primarily, the proliferation of endothelial and smooth muscle cells, along with fibroblasts, and the infiltration of inflammatory cells, underlie the pathological mechanisms (21). Consequently, we hypothesize that factors like hypoxia, infection, autoimmunity, and genetics collectively contribute to abnormal cell proliferation, vasoconstriction, and inflammation, thus fostering PAH, aligning with our study findings.

We identified key genes from both modules through unsupervised cluster analysis and Weighted Gene Co-expression Network Analysis (WGCNA). By intersecting with the differential genes from the previous transcriptome analysis, we pinpointed 130 DISEASE genes. Subsequently, we subjected the hypoxia genes obtained from Genecard to analysis using Disease Ontology (DO), Gene Ontology (GO), and Kyoto Encyclopedia of Genes and Genomes (KEGG). Intersection of the hypoxia gene set with the disease genes yielded 13 hub genes. Next, we constructed a Protein–Protein Interaction (PPI) network for the disease genes, revealing that *CX3CR1*, a hub gene, might indirectly regulate PAH development by modulating *CCL21* and *IL33*. Notably, *CCL21* serves as a crucial marker for predicting PAH development in patients with systemic sclerosis (22) and exhibits significant expression in epithelial lung tissue (23), closely linked to PAH development. Similarly, IL-33 plays a pivotal role in vascular remodeling in PAH (24) by promoting the proliferation of vascular smooth muscle cells through the upregulation of HIF-1α and Vascular endothelial growth factor expression in vascular endothelial cells (25). Consistent with our findings, a mouse study demonstrated that *CX3CR1* deficiency prevents hypoxia-induced PAH by influencing macrophage polarization (26). Consequently, *CX3CR1* emerges as an important biomarker in PAH, mediating vascular and tissue injury (27).

Through immune infiltration analysis, we observed significant differences in immune cell infiltration between PAH patients and normal individuals. PAH patients exhibited heightened infiltration of activated mast cells and memory B cells, while monocytes, naive B cells, and neutrophils were prominently infiltrated in normal subjects. Consistent with prior research, PAH patients displayed increased proportions of activated mast cells and memory B cells, alongside decreased proportions of monocytes, naive B cells, and neutrophils in lung tissues (28–30). PAH and vascular remodeling are strongly linked to various inflammatory cells (31). Mast cells, principal players in allergic reactions, have emerged as crucial contributors to PAH development. They participate in pulmonary vascular remodeling through degranulation, lipid mediator release, and interaction with other inflammatory cells (32). The mast cell-B cell axis notably influences PAH, with mast cells secreting substantial IL-6 amounts that stimulate B cell differentiation into plasma cells, pivotal for PAH and vascular remodeling (33). B cells, in turn, contribute to pathogenic autoantibody production and endothelial cell apoptosis through diverse pathways. In PAH patients, B cell subsets exhibit abnormal distribution, with naive B cell expansion and memory B cell reduction (31). Differential analysis identified hub genes (*FGF2*, *AHR*, *TNFSF10*, *FAS*, *LEPR*, *NEAT1*, *SNHG1*, *CX3CR1*, *ANXA1*, *NAMPT*, *BACH1*, *DLEU2*, and *TSLP*) predominantly highly expressed in the normal group, with most under-expressed in memory B cells and over-expressed in naive B cells. This differential expression pattern suggests a protective role for hub genes.

Analysis of single-cell results showed that features were significantly enriched on macrophages, monocytes, and smooth muscle cells. A prominent pathological feature of PAH is the infiltration of peripheral inflammatory cells, including neutrophils, macrophages, dendritic cells, mast cells, T cells and B cells (31). Among them, the accumulation of macrophages is an important feature of vascular remodeling in PAH, and the activation of macrophages and the synergistic effect with other immune cells are crucial in the occurrence of PAH (34, 35). The characteristic changes of PAH, such as accumulation of inflammatory cells, oxidative stress and proliferation of vascular cells, are related to endothelial cell dysfunction (36). The interference and connection between macrophages, smooth muscle cells and endothelial cells is of great significance for the occurrence and development of PAH (35).

Accurate early diagnosis of Pulmonary Arterial Hypertension (PAH) is crucial, as its progression to later stages can lead to right heart failure and serious health consequences. In recent years, machine learning techniques have gained significant attention for their potential in disease prediction. However, the challenge remains in successfully applying effective machine learning methods to clinical practice while ensuring high accuracy. Specifically, the selection of algorithms is often influenced by personal preferences and inherent biases among researchers (37, 38). To address these issues, this study adopted a systematic approach, integrating twelve different machine learning algorithms and their 113 combinations, and conducted a comprehensive comparison of their diagnostic performance to identify the optimal model, thereby reducing subjective biases in the selection process. Through validation on a training set and two independent test sets, the Naive Bayes model was ultimately identified as the best diagnostic tool. Notably, compared to two other PAH diagnostic models mentioned in the literature, our model relies on a smaller number of genetic markers (39, 40). This not only simplifies the application process but also reduces the complexity of actual implementation, making it easier to achieve clinical translation. By optimizing the balance between accuracy and clinical feasibility, our model has the potential for wider application in clinical environments, enhancing the practicality and accessibility of PAH diagnosis.

This study successfully identified three potential biomarkers for pulmonary arterial hypertension (PAH), namely *AHR*, *FAS*, and *FGF2*, through the application of machine learning strategies. Among these, *AHR* and *FAS* emerged as the most significant. *AHR* and *FAS*, which are closely related to PAH, were identified by constructing a diagnostic prediction model. *AHR*, the gene encoding the aryl hydrocarbon receptor, is widely expressed in vascular endothelial cells. The classical signaling pathway is the genomic pathway, which starts in the cytosol, exists in a latent form, is activated by ligands, and then transfers to the nucleus to regulate gene expression after ligand binding. Including cytochrome P450 family 1 subfamily A member 1 (CYP1A1), cytochrome P450 family 1 subfamily A member 2 (CYP1A2), cytochrome P450 family 1 subfamily B member 1 (CYP1B1), TCDD-induced aggregation (ADP-ribose) polymerase (TIPARP) and aryl hydrocarbon receptor repressor (AHRR) are also closely related to the epidermal growth factor receptor signaling pathway, JAK/STAT pathway and NF-κB family signaling pathway (41–43). *FAS* encodes the death receptor CD95. The death receptor CD95 belongs to the tumor necrosis factor receptor family. Its classical signaling pathway is the apoptosis signaling pathway combined with Fals, which is involved in the process of apoptosis. It also has non-apoptotic signaling pathways that maintain inflammation, regulate immune cell homeostasis and induce cell migration (44, 45). *AHR* and *FAS* may affect the occurrence of PAH by affecting the above pathways. Furthermore, we found that *AHR* and *FAS* were closely associated with monocytes, macrophages and CD8 T cells. Combined with the immune infiltration results, it is not difficult to find that the occurrence of *AHR* and PAH is related to monocytes and macrophages. Monocytes develop and differentiate into phagocytes and dendritic cells after entering tissues or organs, and this process is controlled by *AHR* (46). Macrophages accumulate around blood vessels and cause vasoconstriction, increased vascular permeability and cell proliferation by interfering with immunomodulatory mechanisms, thus promoting the occurrence of PAH, and changes in the microenvironment of phagocytes are an important cause of PAH pathology (35, 47). From this we infer that *AHR* affects PAH development by affecting the expression of macrophages.

To ensure the accuracy of these results, we validated the three genes determined in three independent validation cohorts, further confirming their effectiveness as biomarkers for PAH. However, when we analyzed the expression of these three genes in different datasets, we observed that *FAS* and *FGF2* were both upregulated genes in all three validation cohorts. This phenomenon may be due to the fact that our dataset includes samples from 8 patients with pulmonary fibrosis. PAH secondary to pulmonary fibrosis has unique gene expression characteristics and pathophysiological mechanisms different from other types of PAH (48). Nevertheless, both high expression and low expression of these genes are closely related to the occurrence and development of PAH. We systematically reviewed the existing literature and found supporting evidence (49, 50). Furthermore, in this study, the area under the receiver operating characteristic curve (AUC) of the overall model and the individual gene validation in each dataset was excellent, further validating the reliability and predictive ability of the model.

GSEA analysis of *AHR* and *FAS* showed that the pathways in the low expression group of *AHR* and *FAS* were enriched in basal cell carcinoma, calcium signaling pathway, cardiac muscle contraction, etc. All functions were enriched in G-protein coupled receptor activity. Among them, calcium signaling pathway and G-protein coupled receptor activity play an important role in the occurrence, progression and prognosis of PAH (51, 52). It can be concluded that *AHR* and *FAS* inhibit the development of PAH by affecting the above pathways. In addition, the function of *FAS* low expression group is also enriched in ion channel complex, and the disorder of ion channel can also promote endothelial dysfunction and lead to PAH (53). However, in the existing literature reports, we found that aryl hydrocarbon receptors were more often used as risk factors for PAH (54). Takeshi Masaki’s research indicates that the regulatory mechanism of *AHR* in PAH is different from our research findings (55). We hypothesize that the systemic *AHR* knockout model used by Masaki et al. may have triggered a series of more widespread systemic effects that obscured the specific role of *AHR* in certain immune cells. As a multifunctional nuclear receptor, *AHR* may exhibit diverse biological effects in different cell types (56). Therefore, local or cell-specific *AHR* functions may play a more refined role in the development of PAH. To fully understand the role of *AHR* in PAH, future studies should consider using cell-type-specific knockout models to more accurately assess the function of *AHR* in different immune cells and its impact on PAH.

The exact mechanism of the *AHR* on PAH remains unclear, our study suggests that environmental conditions may suppress *AHR* gene expression in patients with PAH, influencing the biological processes and pathways involved in PAH development. Individual genetic backgrounds may further modulate *AHR* gene expression, impacting susceptibility to PAH. *FAS* is similar.

Finally, we conducted Mfuzz clustering analysis, revealing that clusters 13 and 43 were closely associated with *AHR*. KEGG analysis of these clusters unveiled their association with TNF and phosphatidylinositol signaling systems. These systems are intricately linked with inflammation, proliferation, and migration of pulmonary artery vascular smooth muscle cells, contributing to vascular remodeling (57, 58). Moreover, GO analysis of clusters 13 and 43 indicated enrichment in pathways regulating cell proliferation and inflammatory factors. The proliferation and migration of pulmonary vascular smooth muscle cells are pivotal mechanisms underlying PAH development. Inflammatory factors serve as key drivers of vascular smooth muscle cell proliferation and remodeling (54). Alterations in the *AHR* and *FAS* expression level may thus impact the development of PAH by modulating the expression of cell growth factors and influencing the balance of angiogenic and apoptotic processes, thereby affecting vascular structure and function.

Our study has several limitations. Firstly, the analyses relied on the GEO database, which has a limited sample size, potentially introducing bias. Additionally, our study lacks sufficient experimental validation of the identified genes, highlighting the need for further gene function validation experiments to confirm their viability as diagnostic markers.

## 5 Conclusion

We constructed a diagnostic model for pulmonary arterial hypertension (PAH) based on genes related to cuproptosis and hypoxia, and identified three key diagnostic markers, *AHR*, *FAS* and *FGF2*. Combined with immune infiltration analysis, single cell analysis and GSEA, the results showed that these genes may affect the progression of PAH by regulating cell proliferation and inflammatory response. This finding not only indicates their potential as novel biomarkers, but also provides new strategic directions for the prevention and treatment of PAH by modulating the expression of *AHR*, *FAS*, and *FGF2*.

## Data Availability

Publicly available datasets were analyzed in this study. This data can be found here: the data were obtained from GEO database with corresponding numbers GSE15197, GSE33463, GSE113439, GSE22356, and GSE228644. The direct link is https://www.ncbi.nlm.nih.gov/geo/query/acc.cgi.
