# Supplementary material for: A comprehensive analysis of genes associated with hypoxia and cuproptosis in pulmonary arterial hypertension using machine learning methods and immune infiltration analysis: AHR is a key gene in the cuproptosis process
Source: Front Med (Lausanne). 2024 Sep 26;11:1435068. doi: 10.3389/fmed.2024.1435068 (PMC11464361; doi:10.3389/fmed.2024.1435068)
Supplement: Supplementary file 1 [file Data_Sheet_1.docx]

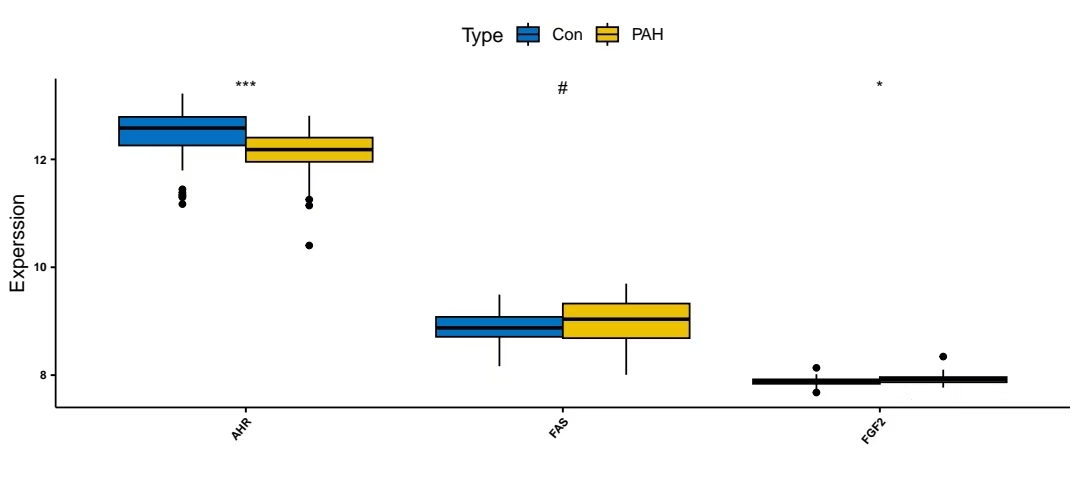
 Figure S1: Expression of three genes in GSE33463.


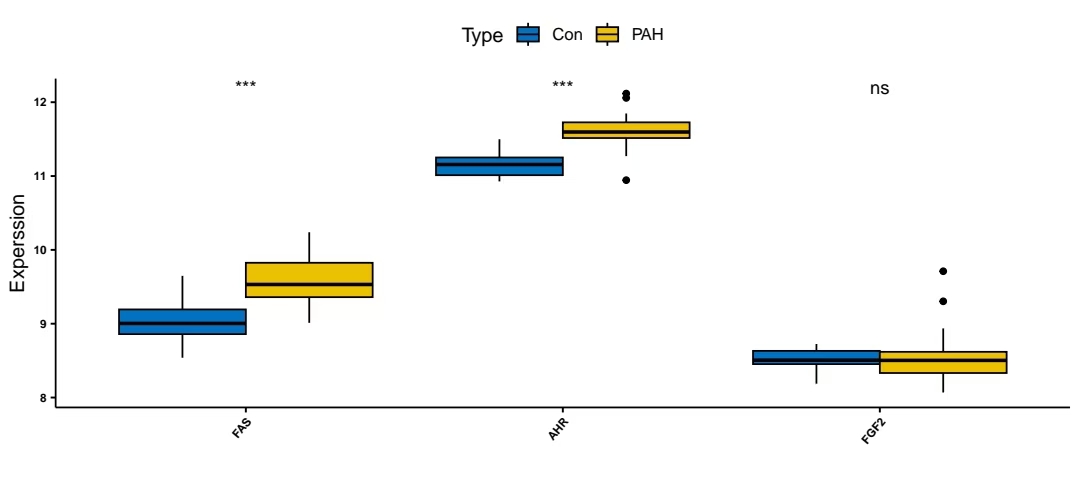


Figure S2: Expression of three genes in GSE113439.


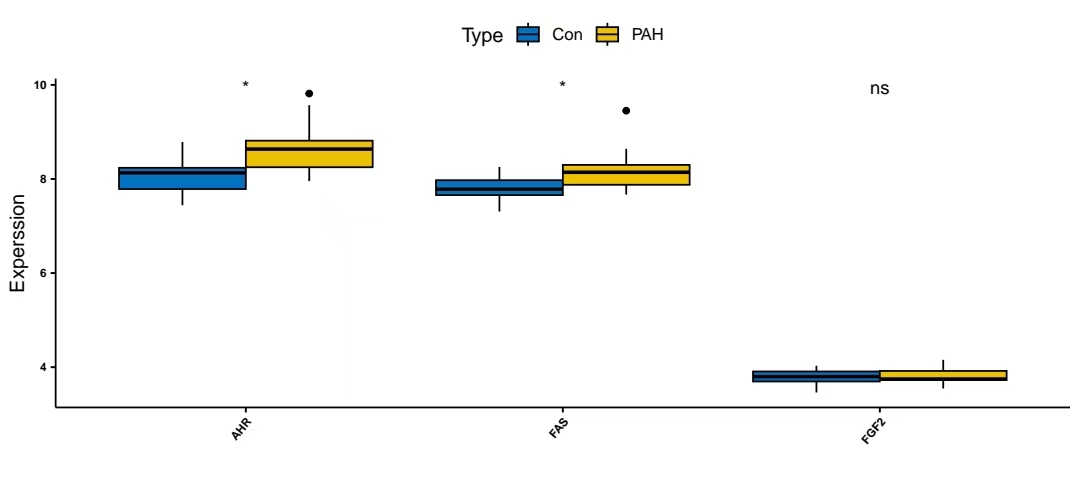
 Figure S3: Expression of three genes in GSE22356.
